# Interaction of Socio-demographic Characteristics on Acceptance of Disability Among Individuals With Physical Disabilities

**DOI:** 10.3389/fpsyt.2021.597817

**Published:** 2021-04-28

**Authors:** Eun-Young Park, Jung-Hee Kim

**Affiliations:** ^1^Department of Secondary Special Education, College of Education, Jeonju University, Jeonju, South Korea; ^2^Department of Clinical Nursing, College of Nursing, The Catholic University of Korea, Seoul, South Korea

**Keywords:** acceptance of disability, employment, education, health, physical disabilities

## Abstract

This study aimed to investigate the interaction of sociodemographic characteristics on acceptance of disability among individuals with physical disabilities (IWPD). Data from the 8th Panel Survey of Employment for the Disabled in Korea (PSED) in the second wave were used. A sample concerning the first phase of disability was extracted using the one-step colony method to extract regions and was stratified based on the type of disability, disability grade, and age. To explore the association between acceptance of sociodemographic characteristics and of disability, we used a general linear model. A significant main effect was observed in employment, health status, degree of help, and subjective economic status. Regarding employment status, acceptance of disability in unemployment of IWPD with less than high school was lower as compared to those with more than high school. We observed that unemployed IWPD with low income or poor health status could be the group with the highest risk for acceptance of disability. Individuals in the low economic group were more religious than those in the high economic one. These findings indicate that specialized intervention programs that consider religion, economic status, employment, education, health, and their interactions would be effective for acceptance of disability. Interdisciplinary team members should consider the individual profiles of these populations and implement suitable support and rehabilitation programs.

## Introduction

Individuals with physical disabilities (IWPD) constitute the largest proportion of all individuals with disabilities. Considering disabilities in only one domain, IWPD in the groups aged 15 and above and in those aged 65 and above were 9.3 and 26.0%, respectively (1). According to the World Health Organization's definition, disability is defined as a result of an interaction between an individual (with a health condition) and contextual factors (environmental and personal). Multifactorial and interactive models have been embraced in understanding disabilities (2, 3).

Although there have been studies analyzing the negative results of disability on an individual's life, such as feeling loss, hopelessness, and depression (4–6), not all people with disabilities experience negative consequences. Successful adaptation to rapid changes in life associated with disabilities has also been reported (7).

There are several mediating variables, such as self-esteem, self-control, coping, and acceptance of disability, which have been known to buffer the negative consequences of disability (8). Among them, acceptance of disability has been consistently reported (9–11). Acceptance of being disabled is a factor that affects the adaptation and community participation of people with disabilities. Acceptance is a critical construct that reflects the extent to which persons cope with their disabilities (12) and is conceptualized as the acceptance of loss (13). Acceptance by people with disabilities has been reported to be a variable affecting successful adaptation (14, 15). Because incorporating their dysfunction and its consequences in their life is the beginning of adjustment (16), acceptance of disability is regarded as a belief about the world and the transformation of values (17). It is considered a final phase of psychosocial adjustment (18) and a critical factor of depression after having a disability (7).

Disability conditions such as acquired disability, multiple disabilities, and chronic pain are also important variables related to disability acceptance (8). Studies have proposed that acceptance is the key component for adjusting to a disabling condition (8). Recently, the acceptance of disability has received more attention in research on physical disabilities, including stroke patients (10), spinal cord injury (7, 9, 17, 19), burn patients (20), patients with colostomies (21, 22), as well as people with hearing and learning disabilities (11, 23, 24). In addition, acceptance is likely to be an important aspect of coping with chronic health conditions among adults experiencing hearing difficulties (11). In the study of parents, psychological acceptance was found to partially mediate the impact of child behavior problems on paternal stress, anxiety, and depression. Acceptance was also a positive predictor of fathers' perceptions of positive gains associated with raising their child with intellectual disability (25). Increased acceptance of the disability extends the belief that one can become a member of society, so accepting it is essential for capacity building and social integration (8).

Although a disability has a negative effect on an individual for a certain period of time, when personal characteristics and social and environmental resources are in place, an objective assessment of the situation can be achieved, and their perception of life can be positively restored (15, 26–28). Several studies have reported associations between demographic characteristics and acceptance of disability. It has been reported to be associated with gender (7, 29) and health status (12, 13). Job accommodation, facilitation of transitional duty, and communication with others are effective components of a disability management program (30). However, there are inconsistent reports that gender, race, and education are not significantly correlated with acceptance of disability (8). Furthermore, the population with disabilities is diverse in age, health conditions, work, and income (31). Therefore, researchers should consider personal assets, including socio-demographic characteristics that increase acceptance of disability.

Knowledge about the source of these differences according to general characteristics is important because it could provide helpful insights to guide therapy or interventions for persons with disabilities. To our knowledge, there is not enough consistent evidence regarding the role of demographic variables in the acceptance of disability. Because of the growing diversity of persons with disabilities who need health and welfare services, we need a better understanding of how the socio-demographic characteristics of IWPD are associated with the acceptance of disability (8), which will be investigated in this study.

This study aimed to investigate the association of variables such as gender, age, marital status, degree of help, religion, economic status, employment, education, health, and their interaction on the acceptance of disability in IWPD.

## Methods

### Data

Data from the second wave of the 8th Panel Survey of Employment for the Disabled in Korea (PSED) were used to analyze how the characteristics of IWPD affect acceptance of disability. These data limited coverage to 4,577 individuals with disabilities aged 15–64. Among the panels, the number of IWPD was 2,250, accounting for 49.2%. This is a representative panel survey that identifies the current status and characteristics of the employment of persons with disabilities. For the sample design, we set the persons registered with the Ministry of Health and Welfare as the target population and adopted the two-phase sampling method, in which the number of extracted regions was adjusted, from which we extracted an appropriate number of samples for each type of disorder, disability grade, and age. Individuals with disabilities were extracted in the first phase sampling through the cluster sampling method within regions, which was stratified by disability type, disability grade, and age that satisfied the target error.

### Measures

#### Acceptance of Disability

Among the 12 items for acceptance of disability used in this panel survey, there were nine extracted by Kaiser et al. (32). Kaiser et al. (32) constructed a questionnaire to measure personal orientation toward disability among the 50 items developed by Linkowski (33). The specific contents of the items were as follows:

First, “I feel satisfied with my abilities, and my disability does not bother me too much.” Second, “Though I am disabled, my life is full.” Third, “It makes me feel very bad to see all the things nondisabled people can do which I cannot.” Fourth, “My disability, in itself, affects me more than any other characteristic about me.” Fifth, “Because of my disability, I am unable to enjoy social relationships as much as I could if I were not disabled.” Sixth, “My disability causes me to think differently about everything.” Seventh, “How a person conducts himself in life is much more important than physical appearance and ability.” Eighth, “Personal characteristics such as honesty and willingness to work hard are much more important than physical ability.” Ninth, “There are many more important things in life than physical appearance.” We adopted a 5-point Likert scale. The third, fourth, and fifth items were coded in reverse. Higher scores indicate a higher acceptance of disability. Cronbach's alpha in this study was 0.85.

#### Age

The age range of the panels in the PSED data ranged from 15 to 64. This study aimed to verify the interaction effect of other variables on age when dividing it as below or above 40 based on general youth age reference.

#### Marital Status

In the PSED, marital status included single, married, or living together, bereavement, and divorce. To analyze the effect of marital status on acceptance of disability, it was divided into marriage or living together. Marital status was categorized according to previous studies (34).

#### Education Level

Education level was recorded as below or above high school. It was categorized according to previous studies (35).

#### Degree of Help

It was measured using a 4-point Likert scale. The degree of help was divided into cases that needed assistance and those that did not. “I do not need it at all” and “I do not need it” were recorded as “not at all.” “It is necessary” and “It is very necessary” were recorded as “necessary.”

#### Subjective Socioeconomic Status

Subjective socioeconomic status was divided into four categories: low, middle, and lower, middle and higher, and high. Subjective socioeconomic status was divided into two categories based on the middle such as “low” and “high.”

#### Health Status

The panel-survey question about general health status used a 4-point Likert scale. Health status was divided into two categories to verify its effects. “Very poor” and “it is not good” were recoded as “bad,” and “good,” and “very good” were recoded as “good.”

#### Religion

Religion was evaluated by recording whether or not they were engaging in religious activities. It was categorized according to a previous study (36).

#### Employment Status

It was divided into employment status and unemployment status. Employment status was categorized according to previous studies (34).

### Data Analysis

Mean differences in sociodemographic characteristics were analyzed using independent *t*-tests. To examine the main effect of the variables on the acceptance of disability and their interaction effects, we used a general linear model (GLM), which is a statistical method that enables regression and variance analysis of categorical factor variables and dependent variables of other independent variables; the method is often used to analyze the mean differences when there is more than one independent variable. The GLM is a generalization of multiple linear regression models and can be applied by the interaction effect that is not sufficiently reflected in the existing regression method. In this study, we employed the GLM univariate model because there was one dependent variable (acceptance of disability). The independent variables were gender, age, marital status, education level, degree of help, subjective socioeconomic status, health status, religion, and employment status. We selected a full factorial design and used Type III sums of squares. We used an analysis to verify the main effect of each variable and examine the interaction effect. This was analyzed by combining two variables; for example, gender × age was used. Statistical analyses were performed using the SPSS 22.0.

## Results

### Socio-demographic Characteristics of the Participants

We analyzed data from 2,250 IWPD. The participants' sociodemographic characteristics according to variables and group differences in acceptance of disability are presented in Tables 1, 2. There were no significant differences between men and women. There were significant differences in other variables, including age, education level, marital status, employment religion, health status, degree of help, and economic status. The mean of acceptance of disability was high for those younger than 40, educated above high school, married, employed, religiously active, well in health status, needing no help, and having a high socioeconomic status.

**Table 1:** The socio-demographic characteristics.

**Category**	***N***	**%**
**Gender**		
Male	1,582	70.3
Female	668	29.7
**Age**		
Below 40	726	32.3
Above 40	1,524	67.7
**Education level**		
Below high school	526	23.4
Above high school	1,724	76.6
**Marital status**		
Married	1,295	57.6
Non-married	955	42.4
**Employment**		
Yes	1,361	60.5
No	889	39.5
**Religion**		
Yes	460	20.4
No	1,728	76.8
**Health status**		
Bad	1,006	44.7
Good	1,233	54.8
**Degree of help**		
Not at all	1,703	75.7
Necessary	547	24.3
**Economic Status**		
Low	1,863	82.8
High	303	13.5

**Table 2:** The differences of acceptance of disability according to socio-demographic characteristic.

**Category**	**Mean**	**SD**	**SE**	***t***	**Mean difference**	**95% CI of mean difference**
**Gender**						
Male	3.20	0.48	0.01	0.953	0.02	−0.02 to 0.06
Female	3.18	0.49	0.02			
**Age**						
Below 40	3.26	0.51	0.02	4.160^**^	0.09	0.05 to 0.13
Above 40	3.17	0.47	0.01			
**Education level**						
Below high school	3.04	0.48	0.02	−8.825^**^	−0.20	−0.25 to −0.16
Above high school	3.24	0.47	0.01			
**Marital status**						
Married	3.27	0.45	0.01	8.526^**^	0.17	0.13 to 0.21
Non-married	3.10	0.51	0.02			
**Employment**						
Yes	3.30	0.45	0.01	12.961^**^	3.26	0.22 to 0.30
No	3.04	0.49	0.02			
**Religion**						
Yes	3.31	0.46	0.02	5.283^**^	0.14	0.11 to 0.18
No	3.17	0.49	0.01			
**Health status**						
Bad	3.04	0.47	0.01	−14.768^**^	−0.29	−0.28 to −0.19
Good	3.33	0.45	0.02			
**Degree of help**						
Not at all	3.25	0.47	0.02	9.948^**^	0.33	1.19 to 0.28
Necessary	3.02	0.49	0.01			
**Economic Status**						
Low	3.17	0.48	0.01	−8.329^**^	−0.25	−0.31 to −0.19
High	3.42	0.50	0.02			

** *p < 0.01*.

### Main and Interaction Effect

**Figure 1:**
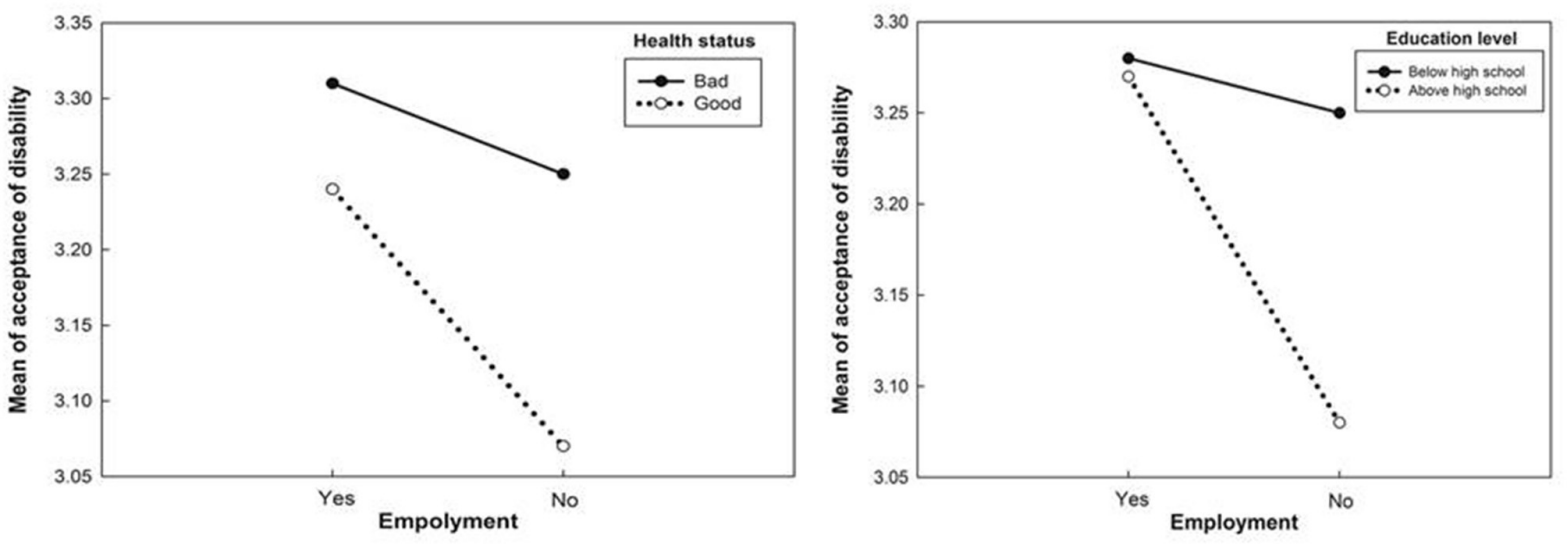
Employment effects of health status and educational level on acceptance of disability.

**Figure 2:**
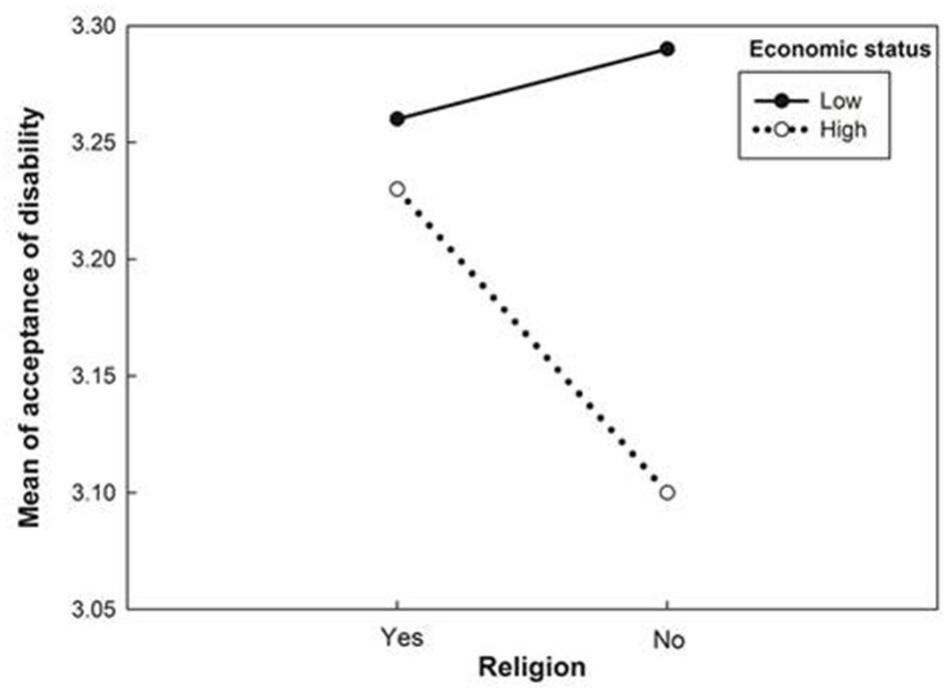
Religion effects of economic status on acceptance of disability.

The main effects of employment status, health status, degree of help, and economic status on acceptance of disability are presented in Table 3. There were no main effects of religion, marital status, age, or education level on acceptance of disability. The interaction effect of employment × health, religion × economic, employment × education on acceptance of disability was significant. Figures 1, 2 show their interaction effects. Although the main effect of education level was not significant, employment and education were significant. Acceptance of disability among IWPD facing unemployment but in good health was more significant than that among employed IWPD in both bad and good health and unemployed IWPD in bad health. Acceptance of disability by IWPD below high school was lower than that for unemployment above high school. The differences in acceptance of disability according to education level were not significant in the employment group; however, the differences were high in the unemployment group. The direction of the regression line was the same; however, the degree of the intercept was different. Similar to the interaction effect of employment and education, the differences in acceptance of disability according to health status were trivial in the employment group; however, the difference was high for bad health status (Figure 1). The interaction effect of religion and economic status is shown in Figure 2. The differences in acceptance of disability according to religion were higher in the low economic group than in the high economic one.

**Table 3:** Main and interaction effect of socio-demographic characteristics.

**Category**	***SS***	***df***	***F***	**Partial Eta Squared**
Gender	0.003	1	0.003	0.017
Employment	1.234	1	1.234^*^	6.233
Religion	0.201	1	0.201	1.013
Marital	0.573	1	0.573	2.892
Age	0.019	1	0.019	0.094
Education	0.455	1	0.455	2.299
Health	1.311	1	1.311^*^	6.624
Help	2.687	1	2.687^**^	13.571
Economic	0.979	1	0.979^*^	4.942
Gender × employment	0.547	1	0.547	2.761
Gender × religion	0.005	1	0.005	0.023
Gender × marital	0.015	1	0.015	0.077
Gender × age	0.218	1	0.218	1.100
Gender × education	0.001	1	0.001	0.006
Gender × health	0.424	1	0.424	2.139
Gender × help	0.194	1	0.194	0.980
Gender × economic	0.086	1	0.086	0.433
Employment × religion	0.058	1	0.058	0.294
Employment × marital	0.121	1	0.121	0.614
Employment × age	0.109	1	0.109	0.551
Employment × education	2.228	1	2.228^**^	11.254
Employment × health	0.776	1	0.776^*^	3.917
Employment × help	0.270	1	0.270	1.362
Employment × economic	0.622	1	0.622	3.143
Religion × marital	0.260	1	0.260	1.314
Religion × age	0.044	1	0.044	0.224
Religion × education	0.147	1	0.147	0.743
Religion × health	0.128	1	0.128	0.646
Religion × help	0.034	1	0.034	0.172
Religion × economic	0.897	1	0.897^*^	4.531
Marital × age	0.308	1	0.308	1.555
Marital × education	0.305	1	0.305	1.539
Marital × health	0.001	1	0.001	0.006
Marital × help	0.581	1	0.581	2.932
Marital × economic	0.118	1	0.118	0.596
Age × education	0.000	1	0.000	0.002
Age × health	0.000	1	0.000	0.001
Age × help	0.002	1	0.002	0.010
Age × economic	0.097	1	0.097	0.492
Education × health	0.060	1	0.060	0.302
Education × help	0.217	1	0.217	1.097
Education × economic	0.045	1	0.045	0.228
Health × help	0.032	1	0.032	0.162
Health × economic	0.125	1	0.125	0.630
Help × economic	0.394	1	0.394	1.989

*SS, sum of square*;

** *p < 0.01*;

* *p < 0.05*.

## Discussion

The aim of this study was to investigate how the socio-demographic characteristics of IWPD are associated with acceptance of disability. The mean difference according to socio-demographic characteristics in the acceptance of disability in IWPD was significant for age, education level, marital status, employment, religion, health status, degree of help, and economic status. In the GLM analysis, a significant main effect was shown in employment, health status, degree of help, and subjective economic status. There was some difference between the mean difference results and the main effect because the GLM could verify the effect of each independent variable while controlling for other variables. This is similar to the covariance analysis procedures. In terms of gender, the mean with difference acceptance of disability results was consistent with previous studies that reported gender differences (7), but the main effect was not consistent. The results of employment status, health status, and functional outcomes were in line with those of previous studies (12, 13).

However, the interaction of socio-demographic characteristics on the acceptance of disability in IWPD was significant for employment × education, employment × health status, and religion × economic status. In terms of employment status, acceptance of disability in unemployed IWPD with less than high school was lower than that of unemployed IWPD with more than high school. A higher level of education and a less severe health status had a more positive acceptance of disability among patients with colorectal cancer (21). In this study, there was less difference in acceptance of disability according to level of education and health status under the employment of IWPD because of the interaction among variables. We found that unemployment of IWPD in low income or bad health status could be associated with the highest risk group for acceptance of disability.

The differences in socio-demographic characteristics according to the presence or absence of a job indicate that it has a moderating effect on accepting the disability. The moderating role of employment status is reinforced. To verify the differences in education level and health status on accepting the disability, the result may depend on the presence or absence of a job, so education level or health status alone cannot determine the influence on accepting the disability.

It could be argued that employment itself provides economic benefits and career development in IWPD (37). In addition, better health status may be related to better functional outcomes and lead to easier employment. Considering that the employment of IWPD would improve the quality of life as well as their income (38), further studies should focus on the characteristics of employment of people with IWPD. Therefore, interventions designed for rehabilitation programs to increase acceptance of disability should address the education level and employment status in IWPD.

The role of religion was higher in the low economic group than in the high economic one. Acceptance of disability has a social aspect because culture or social stigma can affect acceptance of disability (39, 40). Considering that support from the community or outside the family has a positive effect on the acceptance of disability (13), healthcare providers should integrate these variables into rehabilitation programs to improve acceptance of disability in the high-risk group.

In many cultures, stigma can be an obstacle to acceptance of disability (17, 39). Social norms have also been a barrier to the implementation of handicapped-awareness programs for the general population (40), as well as for people with disabilities (41). Thus, increasing the employment and facilitation of support from others, such as religious activities, would be an effective component for a disability management program.

Acceptance of disability has contributed to psychological resources, including hope and posttraumatic growth (17), self-efficacy (41), and physical aspects, such as clinical outcomes. The findings provide a comprehensive understanding of the importance of screening for acceptance of disability and provide useful insights to guide specialized intervention programs that consider religion, economic status, employment, education, health, and their interactions. The obtained results could expand the knowledge that education and employment are important and should be actively applied in integrative labor market policies and rehabilitation programs.

This correlational study has several limitations. Our findings should be generalized with caution because it was impossible to set directional hypotheses and explain cause-effect relationships given the cross-sectional nature of the study design. Despite the use of systematic sampling methods and large-scale panel survey data, there might be variables that could be considered as potential confounders or mediators of other variables in the analysis. Longitudinal research is needed to explain the differences in the acceptance of disability according to general characteristics. In addition, self-reported bias may affect the interpretation of our data, and some of the variables, in particular the degree of help, subjective socioeconomic status, and health status, were subjectively measured. Thus, the validity of our results might have been influenced by the social desirability bias and intrinsic self-reporting bias. Despite these limitations, the current study is the first to apply the GLM methodology to investigate the association of variables such as gender, age, marital status, level of help, religion, economic status, employment, education, health, and their interaction on the acceptance of disability in IWPD.

## Conclusion

This study may contribute to the discussion on the role and importance of general characteristics and their interaction with the acceptance of disability in IWPD. These findings indicate that specialized intervention programs that consider religion, economic status, employment, education, health, and their interactions would be effective for the acceptance of disability. Interdisciplinary members should take into account the individual profiles of this population and apply suitable support and rehabilitation programs.

## Data Availability Statement

The raw data supporting the conclusions of this article will be made available by the authors, without undue reservation.

## Ethics Statement

Ethical review and approval was not required for the study on human participants in accordance with the local legislation and institutional requirements. The patients/participants provided their written informed consent to participate in this study.

## Author Contributions

E-YP and J-HK contributed to conception the study, conducted the statistical analysis and interpretation of the data, and drafted the manuscript. Both authors read and approved the final manuscript.

## Conflict of Interest

The authors declare that the research was conducted in the absence of any commercial or financial relationships that could be construed as a potential conflict of interest.
